# Identification of miRNAs contributing to neuroblastoma chemoresistance

**DOI:** 10.1016/j.csbj.2015.04.003

**Published:** 2015-04-29

**Authors:** Duncan Ayers, Pieter Mestdagh, Tom Van Maerken, Jo Vandesompele

**Affiliations:** a Centre for Molecular Medicine and Biobanking, University of Malta, Msida, Malta; b Manchester Institute of Biotechnology, Faculty of Medical and Human Sciences, The University of Manchester, United Kingdom; c Center for Medical Genetics Ghent, Ghent University Hospital, Ghent, Belgium

**Keywords:** miRNA, Drug, Resistance, Chemoresistance, Neuroblastoma

## Abstract

**Background:**

The emergence of the role of microRNAs (miRNAs) in exacerbating drug resistance of tumours is recently being highlighted as a crucial research field for future clinical management of drug resistant tumours. The purpose of this study was to identify dys-regulations in expression of individual and/or networks of miRNAs that may have direct effect on neuroblastoma (NB) drug resistance.

**Methods:**

Individual subcultures of chemosensitive SH-SY5Y and UKF-NB-3 cells were rendered chemoresistant to doxorubicin (SH-SY5Y, UKF-NB-3) or etoposide (SH-SY5Y). In each validated chemoresistance model, the parental and subcultured cell lines were analysed for miRNA expression profiling, using a high-throughput quantitative polymerase chain reaction (RT-qPCR) miRNA profiling platform for a total of 668 miRNAs.

**Results:**

A unique expression signature of miRNAs was found to be differentially expressed (higher than 2-fold change) within all three NB chemoresistance models. Four miRNAs were upregulated in the subcultured chemoresistant cell line. Three miRNAs were found to be downregulated in the chemoresistant cell lines for all models.

**Conclusions:**

Based on the initial miRNA findings, this study elucidates the dys-regulation of four miRNAs in three separate NB chemoresistant cell line models, spanning two cell lines (SH-SY5Y and UKF-NB-3) and two chemotherapeutic agents (doxorubicin and etoposide). These miRNAs may thus be possibly linked to chemoresistance induction in NB. Such miRNAs are good candidates to be novel drug targets for future miRNA based therapies against aggressive tumours that are not responding to conventional chemotherapy.

## 1 Background

Undoubtedly, one of the most frequently occurring malignancies in childhood is neuroblastoma (NB) [1], in most cases affecting children under the age of five [2]. Neuroblastoma is a tumour that develops in the embryonic stage [3] and derives from primordial cells of the sympathetic nervous system known as neural crest cells [4]. These progenitor cells cease to differentiate and mature, which is the natural course of events in neural crest cells en-route to the development of the sympathetic nervous system in the embryo [5]. The tumourigenesis from these progenitor cells is thought to be due to the formation of self-regenerating tumour stem cells that have the ability to produce a distinct range of different NB cell lineages according to their histology [6].

The clinical manifestations and degree of severity of NB may be highly varied [7]. The initial phase of the condition is the presence of a painless lump on the abdomen, neck or chest of the child [8]. However, the tumour usually undergoes metastasis and the child consequently presents symptoms due to the NB tumour acting as a space-occupying lesion in the areas of the body that are affected. In order to aid clinicians and oncologists to determine the severity of NB in the individual patient and consequently implement more bespoke treatment strategies, an International Neuroblastoma Staging System (INSS) [9] was devised, based on the degree of NB metastasis and microscopic analysis of the afflicted tissues of the patient. This staging system has proved to be of immense value and is still presently being applied for neuroblastoma diagnosis.

In overview, patients diagnosed with stage 1 or 2 NB conditions are mainly subjected to surgery for excising the tumour mass, without the use of chemotherapeutic cycles or other treatments [10]. However, patients with stage 3 or 4 NB are at high risk and thus tumour de-bulking/removal surgery is combined to other treatment strategies such as chemotherapy (with or without bone marrow transplantation) and low-dose radiotherapy [10].

Unfortunately, the emergence of chemoresistance within tumour cells of solid tissues is one of the main reasons for treatment failure and relapse in patients suffering from metastatic cancer conditions [11]. Resistance of the tumour cell to chemotherapeutic agent exposure may be innate, whereby the genetic characteristics of the tumour cells are naturally resistant to chemotherapeutic drug exposure [12]. Alternatively, chemoresistance can be acquired through development of a drug resistant phenotype over a defined time period of exposure of the tumour cell to multiple chemotherapy combinations [11,12]. Apart from other biological and genetic factors influencing the chemoresistance properties of such tumours, the role of non-coding RNAs such as micro RNAs (miRNAs) [13,14] in the induction of such a phenotype is rapidly being recognised [15–19].

Within the context of NB, there already exist links between miRNA dysregulated expression patterns and NB clinical severity [20]. However, scientific literature provides limited studies relating to the identification of miRNAs directly contributing to NB chemoresistance, with one of them being *miR-17-5p* [21]. This study revealed that the exposure of chemotherapy resistant NB murine tumour models to antagomirs for miR-17-5p resulted in an increase in prognosis, due to enhanced apoptosis and cell cycle arrest effects [21]. In addition, miR-21 is a known oncomir and was found to be over-expressed in cisplatin resistant NB cell lines [22]. Another study has also identified miR-204 as a potentially important miRNA that conveys suppressive properties against the chemoresistance phenotype in NB [23].

## 2 Methodology

The purpose of this study was to identify and validate (if any) specific miRNAs that could be directly involved in the exacerbation of chemoresistance properties in NB cell lines. The utilisation of RT-qPCR based miRNA profiling can nowadays be employed for serving such research objectives.

### 2.1 Description of NB cell line chemoresistance models

Eight separate NB cell line models were implemented in this study. Each model consisted of two cell lines, a parental chemosensitive together with a sub-cultured cell line that was rendered chemoresistant to a single conventional chemotherapeutic agent through repeated exposure (see Table 1).

### 2.2 Validation of chemosensitivity status for NB cell lines

Prior to miRNA profiling of all NB cell lines, validation of their chemosensitivity status was paramount to ensure that the subcultured chemoresistant cell line in each model retained its drug resistance properties over several culturing passages without further drug exposure.

The method adopted for validating chemoresistance status in each model consisted of luminescence-based cell viability analysis, through the utilisation of the Cell Titer Glo assay [Promega, USA] on a Fluorostar Optima luminescence plate reader platform [BMG, Germany], according to the manufacturer's protocol. Due care was applied in maintaining a seeding cell population across all culture wells utilised in the functional assay, in order to avoid false positive viability readings.

#### 2.2.1 Cell culturing conditions and harvesting

The growth medium utilised for culturing of all cell lines in this study consisted of RPMI 1640 [Gibco, USA] treated with 10% foetal calf serum [Gibco, USA] and 1% by volume of 1:1 penicillin/streptomycin mix 10,000 U/mL, kanamicin 10 mg/mL and l-glutamate 200 mM [Gibco, USA] respectively. Prior to a three-minute step within T25 cell culturing flasks [Corning, USA], versene solution [Gibco, USA] was used for washing all adherent cells within the culture flasks. The trypsin solution utilised consisted of 0.05% trypsin with EDTA [Gibco, USA]. Harvesting of cell suspensions for RNA extraction was performed by adding 0.7 mL Qiazol solution [Qiagen, Germany].

#### 2.2.2 NB chemoresistance model cell viability assay

For each chemoresistance model investigated in this study, both the parental chemosensitive and subcultured chemoresistant NB cell lines were grown in culture and simultaneously harvested and transferred to a sterile 50 mL tube [BD Bioscience, USA]. The cell population density for each cell line was counted by extraction of a 20 μL sample (following vigorous pipette mixing) and analysed on a Cellometer Auto T4 cell counter platform [Nexcelom Bioscience, USA] according to manufacturer's protocol. Both cell cultures where then diluted as necessary with sufficient growth medium to produce a cell culture with a cell population density of approximately 10,000 cells/95 μL. Consequently, 95 μL aliquots from each cell line were transferred (following vigorous pipette mixing) to an opaque Nunclon 96 well microtitre plate [Nunc, USA].

For each cell line, 18 wells were utilised for consequent drug treatment, and nine wells were to serve as non-treated NB negative controls (NB cell culture only, with no drug exposure). A further six wells were treated with 195 μL growth medium only, in order to act as negative controls (drug exposure only). All remaining wells were treated with 200 μL growth medium. Finally, the microtitre plate was placed in an incubator at 37 °C and 5% CO_2_ for 24 h, to allow cell adhesion on well surface.

Following the 24-hour incubation, the microtitre plate containing adherent cells was retrieved and treated with the appropriate chemotherapeutic agents for each chemoresistance cell line model. The varying drug dilutions (see Table 2) were prepared on the same day when used for treatment, using filter sterilised water [Sigma, USA] within the confines of a Class II laminar flow cabinet. All light sensitive drug dilutions were also covered in aluminium foil until required.

Once retrieved from the incubator, the plate was placed inside a laminar flow cabinet and all wells designated for treatment with the appropriate chemotherapeutic agent were exposed. A 5 μL aliquot of the appropriate drug and dose was pipetted, following vigorous pipette mixing of the drug solution, into the appropriate triplicate wells. An extra 5 μL aliquot of each drug dose was applied to a single well containing only growth medium, in order to obtain background luminescence data from a drug/growth medium solution for future use following cell viability analysis. The plate was then re-incubated for a further 36 h.

Following this time period, the plate was collected and a cell viability assay was performed on all wells, by using the Cell Titer Glo assay [Promega, USA] according to manufacturer's protocol.

#### 2.2.3 Data analysis and statistical analysis

Following the assay, the Excel sheet containing all raw luminescence data was collected for analysis, with outliers being identified and discarded. The chemosensitivity profiles, based on the cell viability analysis data set results, were plotted by application of the Graph Pad Prism software package [GraphPad Software Inc., USA]. No detailed statistical analysis was performed on the resultant data sets as long as the inhibiting concentration for 50% cell viability (IC50) demonstrated distinct values, reflecting the chemosensitivity profiles.

### 2.3 NB cell line miRNA profiling

#### 2.3.1 Cell line harvesting and lysis

All cell lines from each validated NB chemoresistance model were cultured and harvested accordingly. Following trypsin incubation step, each T25 flask was subjected to horizontal mechanical shock, necessary for dislodging all cells from the flask surface. The resultant cell suspension from each flask was transferred to an individually labelled, sterile 15 mL collection tube [BD Biosciences, USA] and centrifuged at 1500 rpm for five minutes to allow all cells to form a pellet at the base of the collection tube. The supernatant from each collection tube was aspirated by disposable, sterile glass pipette with due care to avoid inadvertent aspiration of cell pellet. Consequently, the remaining cell pellet was treated with 700 μL of Qiazol solution [Qiagen, Germany] in order to induce cell lysis. This step was performed within the confines of a fume cupboard and thorough pipette-mixing was applied for ensuring a resultant homogenous cell lysate suspension. Immediately after cell lysis induction, all cell lysate suspensions were snap frozen in liquid nitrogen solution and transferred to a − 80 °C freezer until RNA extraction was performed.

#### 2.3.2 miRNA extraction procedure

All NB cell line lysates were allowed to thaw to room temperature prior to miRNA extraction. Consequently, all lysates were treated with the miRNeasy [Qiagen, Germany] miRNA extraction protocol, within the confines of a fume cupboard. The resultant 30 μL volume of extracted miRNA from each NB cell line lysate was quantified through utilisation of the Nanodrop UV spectrophotometry platform [Nanodrop Technologies, USA]. The extracted miRNA samples were then stored at − 80 °C until further use. The RNA quality was not tested following extraction, as previous experience from earlier studies repeatedly demonstrated that the RNA quality derived from NB cell lines was always of an excellent nature (with RIN values above 9.0), thus such a step was not performed.

#### 2.3.3 RT-qPCR based miRNA profiling

All miRNA samples were allowed to thaw prior to further treatments. The protocol utilised in this step was a validated, high throughput RT-qPCR based miRNA profiling method [30]. In summary, miRNA samples were diluted to a standardised concentration and initially subjected to a megaplex reverse transcription protocol [30]. This was followed by a pre-amplification step and eventual qPCR analysis [30].

#### 2.3.4 Data normalisation and analysis

All raw Cq values obtained from each individual run were corrected by inter-run calibrators (five individual small nucleic RNA assays — RNU24, RNU44, RNU48, RNU6B, U6 snRNA; two technical replicates/assay/plate) and consequently normalised against the average Cq value obtained from the total quantity of miRNAs assayed within the same run [27]. The normalised Cq expression data for each miRNA was compared between the constituent chemosensitive and chemoresistant cell lines for each chemoresistance model. All miRNAs found to be dysregulated within the chemoresistant cell line, following the adoption of a +/− 2 × linear fold change cut-off value, were deemed to be putative chemoresistance miRNAs within the individual chemoresistance model. This nominal cut-off value is subjective and was deemed to be acceptable to attain a legitimate short list of miRNAs having a tangible expression dysregulation profile of sufficient weight as to lead to an overall shift in chemosensitivity profile (in this case, see Discussion section).

Ultimately, all miRNAs dysregulated in all three chemoresistance models' miRNA shortlists were identified and selected for further investigation.

#### 2.3.5 Multiplex RT-qPCR for putative chemoresistance miRNAs

The putative miRNAs identified from miRNA profiling to be dysregulated in the chemoresistant cell lines were further analysed by multiplex RT-qPCR.

Taqman miRNA RT kit [ABI, USA] was utilised for the reverse transcription step. Reverse transcriptase (RT) primers specific for the relevant target miRNAs [ABI, USA] were diluted (5 nmol) with 250 μL nuclease free water [Sigma, USA], and 10 μL aliquots from each individual primer solution were pooled in a 1.5 mL Eppendorf tube. The primer pool was then diluted with nuclease free water or concentrated by vacuum centrifugation in order to obtain a final volume equivalent to 20% of the total RT reaction volume utilised in this step. The RT reaction volume (per well) consisted of 4 μL stem-loop primer pool, 0.4 μL of 100 nM dNTPs, 4 μL MultiScribe reverse transcriptase [Applied Biosystems, USA], 2 μL of 10 × RT buffer, 0.25 μL RNase inhibitor [Applied Biosystems, USA] and 0.65 μL nuclease free water. The total reaction volume was then placed in a thermal cycler and subjected to 30 min at a temperature of 16 °C, followed by 30 min at 42 °C, 1 s at 50 °C, 5 min at 85 °C and 5 min at 4 °C respectively. The finalised reaction volume was diluted by a factor of five, prior to RT-qPCR step.

The TaqMan® miRNA assay for RT-qPCR quantification was utilised in this step for all miRNA assays. Assays for hsa-mir-99b, hsa-mir-125a and hsa-mir-425 were also prepared as reference miRNAs for post-run data normalisation and analysis. All miRNA assays for each cell line sample cDNA were performed in triplicate, with water — containing negative controls. The reaction volume (per well) contained 2.5 μL TaqMan Master mix, 0.125 μL TaqMan miRNA probe and primers, 2 μL of sample cDNA and 0.375 μL nuclease free water. The cycling protocol was run on the ABI 7900HT qPCR platform [ABI, USA] and consisted of an initial holding stage of 95 °C for 10 min, followed by 40 cycles of 95 °C for 15 s/60 °C for 1 min, ending with a final holding stage of 37 °C for 5 min. Following the qPCR run, the data was exported and analysed, including statistical analysis, on the qBasePlus software package [Biogazelle, Belgium].

## 3 Results

### 3.1 Validation of chemosensitivity status for NB cell lines

Prior to performing miRNA profiling for each individual cell line within the varying NB chemoresistance models, it was essential to confirm the chemosensitivity profiles. This was implemented by performing a luminescence based cell viability assay, following a pre-determined exposure period by the cell lines to the relevant chemotherapeutic agent. However, the luminescence detection platform also required preliminary analysis to confirm optimum performance.

**Fig. 1:**
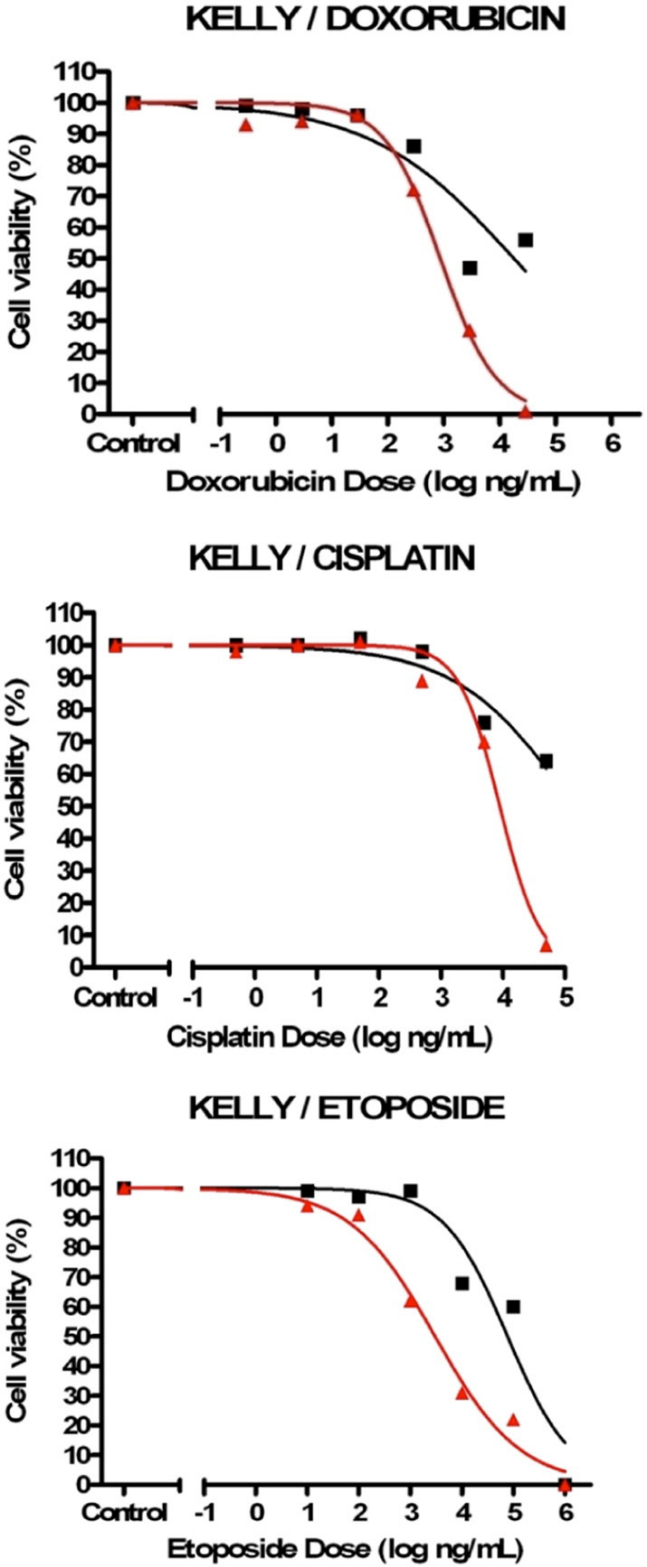
Results for cell viability analysis of Kelly NB chemoresistance models (n = 1, three technical replicates/data point, SEM not illustrated). In all three assays the chemosensitivity status for each component NB cell line was not suitable for inclusion in the miRNA profiling study (black — chemosensitive parental NB cell line; red — chemoresistant NB cell line).

**Fig. 2:**
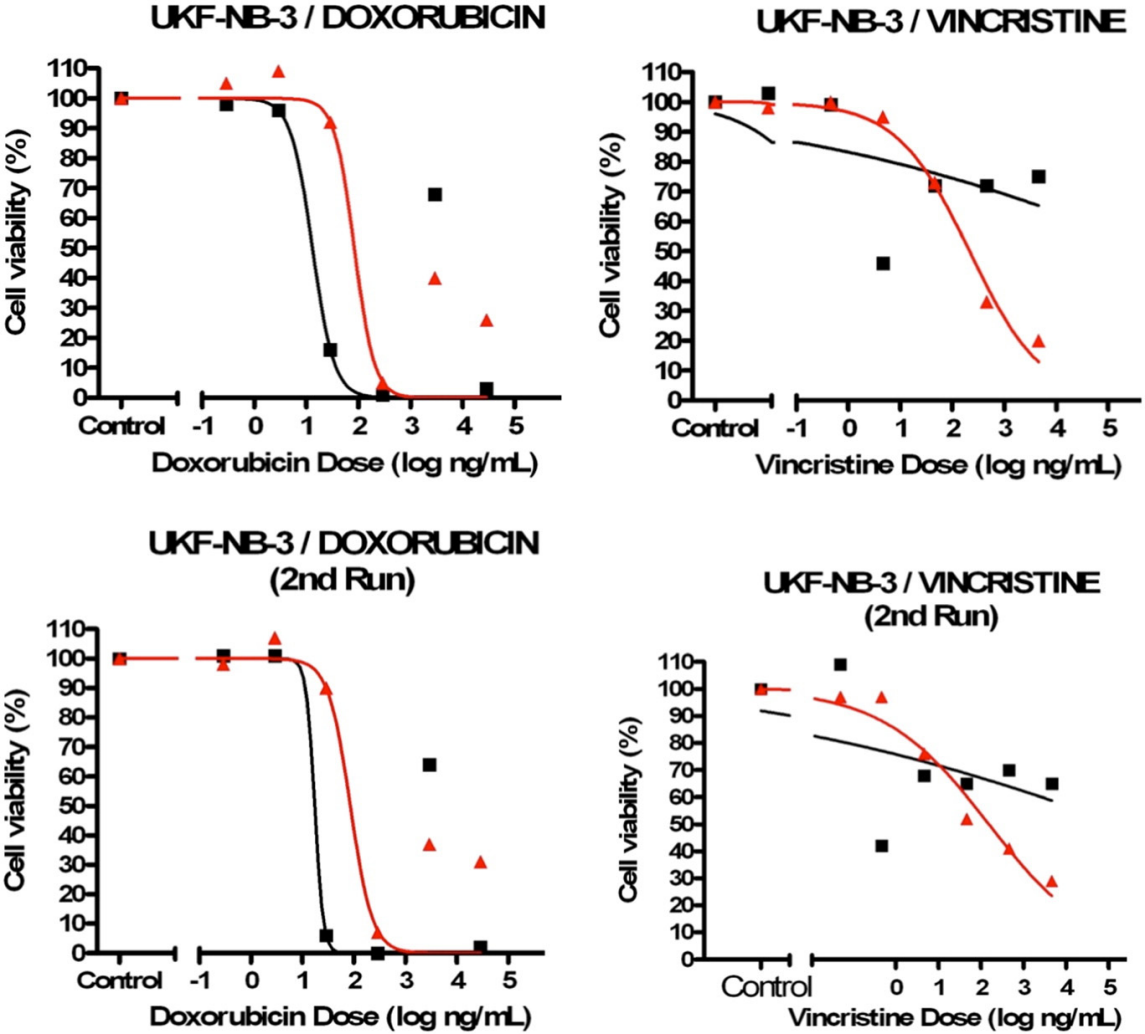
Results for cell viability analysis of UKF-NB-3 NB chemoresistance models (two separate runs, n = 1, three technical replicates/data point, SEM not illustrated). The doxorubicin chemoresistance model was included in the miRNA profiling study due to a 10-fold linear change in the IC50 dose for both constitutive cell lines. However, at elevated doxorubicin doses a reproducible, artificial increase in cell viability was noticed within this NB chemoresistance model. The UKF-NB-3/vincristine NB chemoresistance model was not included in the study due to incongruent chemosensitivity profiles for each constituent NB cell line (black — chemosensitive parental NB cell line; red — chemoresistant NB cell line).

**Fig. 3:**
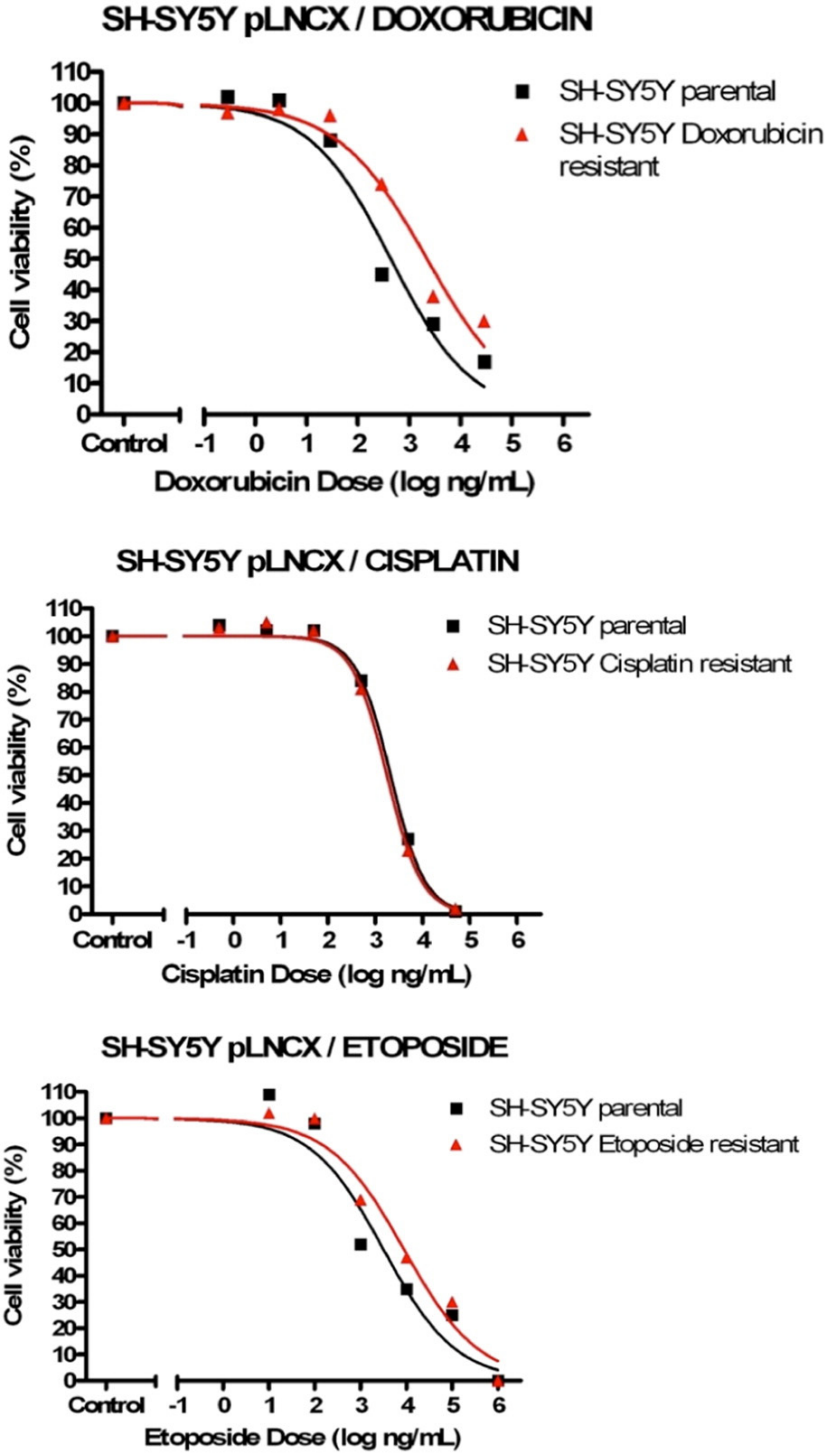
Results for cell viability analysis of SH-SY5Y NB chemoresistance models (n = 1, three technical replicates/data point, SEM not illustrated). The cisplatin chemoresistance NB model was not included in the study due to lack of a suitable chemosensitivity profile by the predicted chemoresistant NB cell line component for this model.

**Fig. 4:**
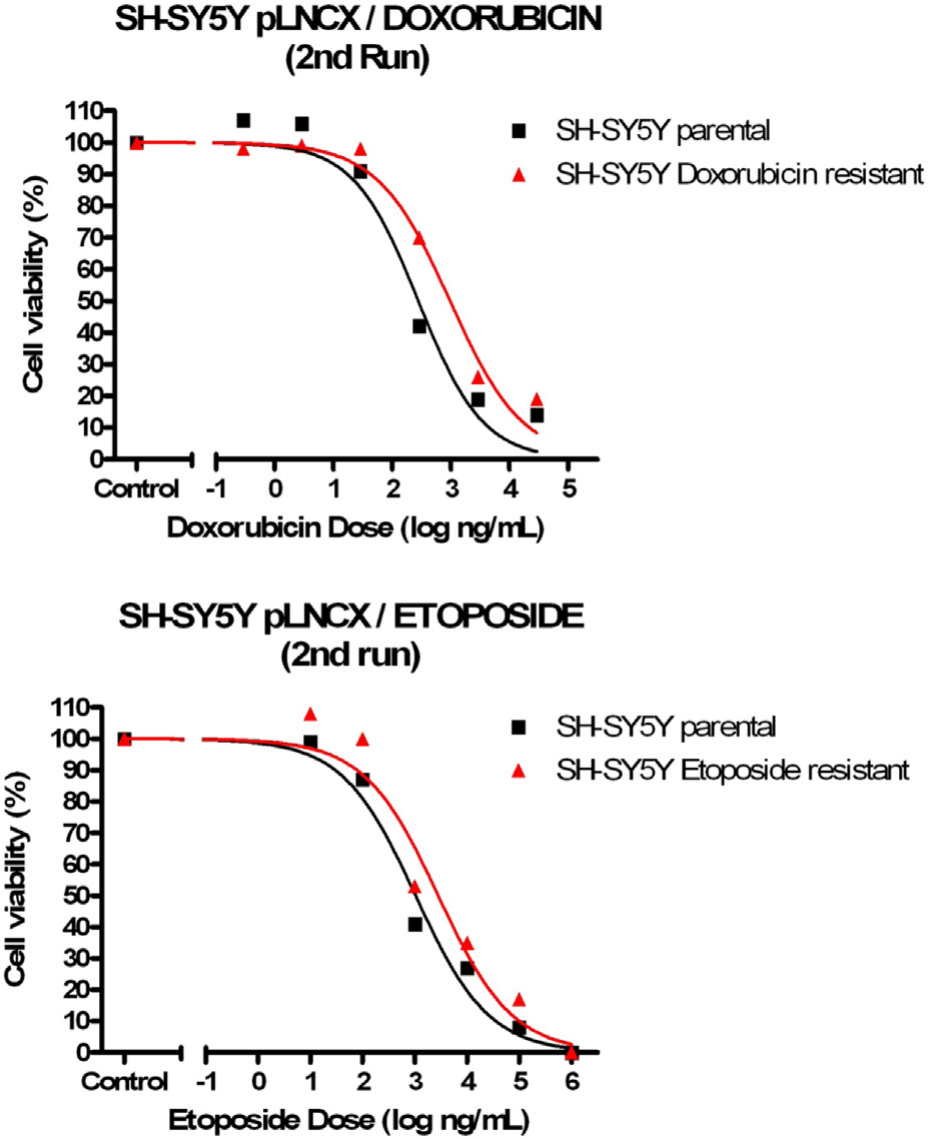
Cell viability analysis results (2nd run, n = 1, three technical replicates/data point, SEM not illustrated) for re-confirmation of chemosensitivity profiles for each constituent NB cell line chemoresistance model.

From the eight NB chemoresistance cell line models available, cell viability analyses only confirmed three models to still have the necessary chemosensitvity profiles required for investigating miRNA expression profiling (see Figs. 1–4). The three validated models (SH-SY5Y/ETOPO, SH-SY5Y/DOXO, UKF-NB-3/DOXO) were analysed by two individual cell viability assays to ensure chemosensitivity profiles.

Each NB chemoresistance cell line model was analysed to confirm chemosensitivity status of its constituent cell lines. The cell lines that confirmed chemosensitivity status were re-analysed with a second, identical cell viability analysis in order to ascertain such status prior to miRNA profiling.

Following the set of cell viability assays on each NB chemoresistance model, only three out of the initial eight cell line models were confirmed to have maintained their chemosensitivity profiles, namely the SH-SY5Y/DOXO, SH-SY5Y/ETOPO and UKF-NB-3/DOXO NB chemoresistance models. The extracted RNA from constituent cell lines, from each of the three cell line models, were consequently eligible for miRNA profiling.

### 3.2 NB cell line miRNA profiling

**Fig. 5:**
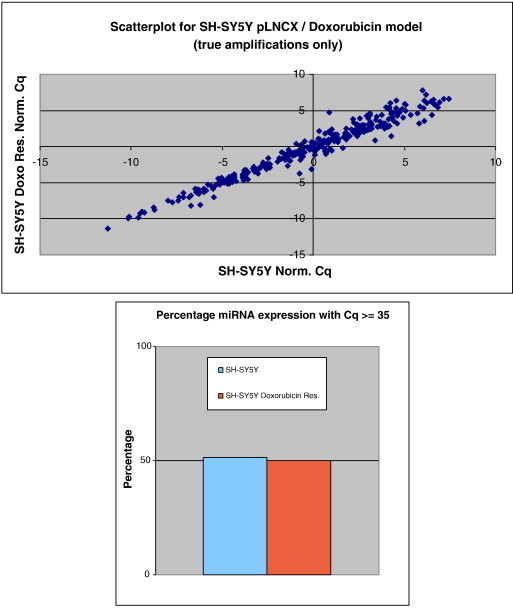
Scatterplot of miRNA expression for SH-SY5Y/DOXO cell line model constituents. This highlights only the marginal variation in the percentage of miRNAs that are actually expressed within the individual cell lines.

**Fig. 6:**
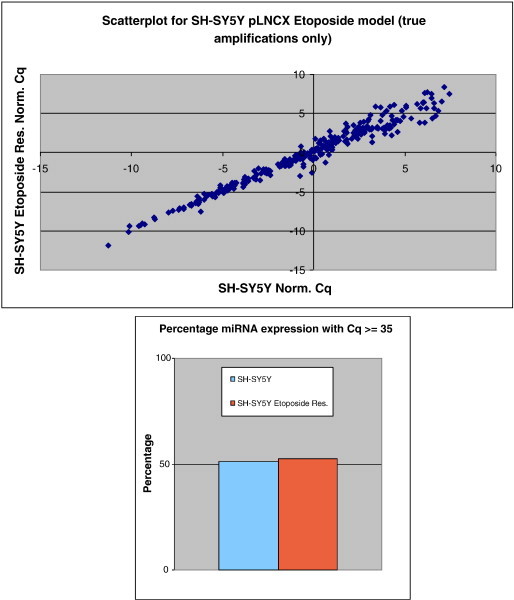
Scatterplot of miRNA expression for SH-SY5Y/ETOPO cell line model constituents. This highlights only the marginal variation in the percentage of miRNAs that are actually expressed within the individual cell lines.

**Fig. 7:**
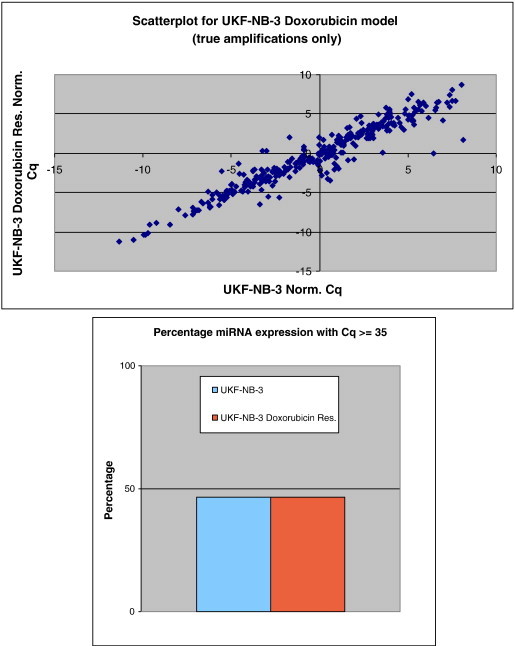
Scatterplot of miRNA expression for UKF-NB-3/DOXO cell line model constituents. This highlights insignificant variation in the percentage of miRNAs that are actually expressed within the individual cell lines.

The expression profiling of 668 miRNAs for each constituent cell line of the three validated and selected NB chemoresistance cell line models demonstrated that approximately 50% of all miRNAs are expressed above the 35 Cq value (see Figs. 5–7). Additionally, the expression scatterplot profiles highlight that overall miRNA expression is cell line dependent.

**Fig. 8:**
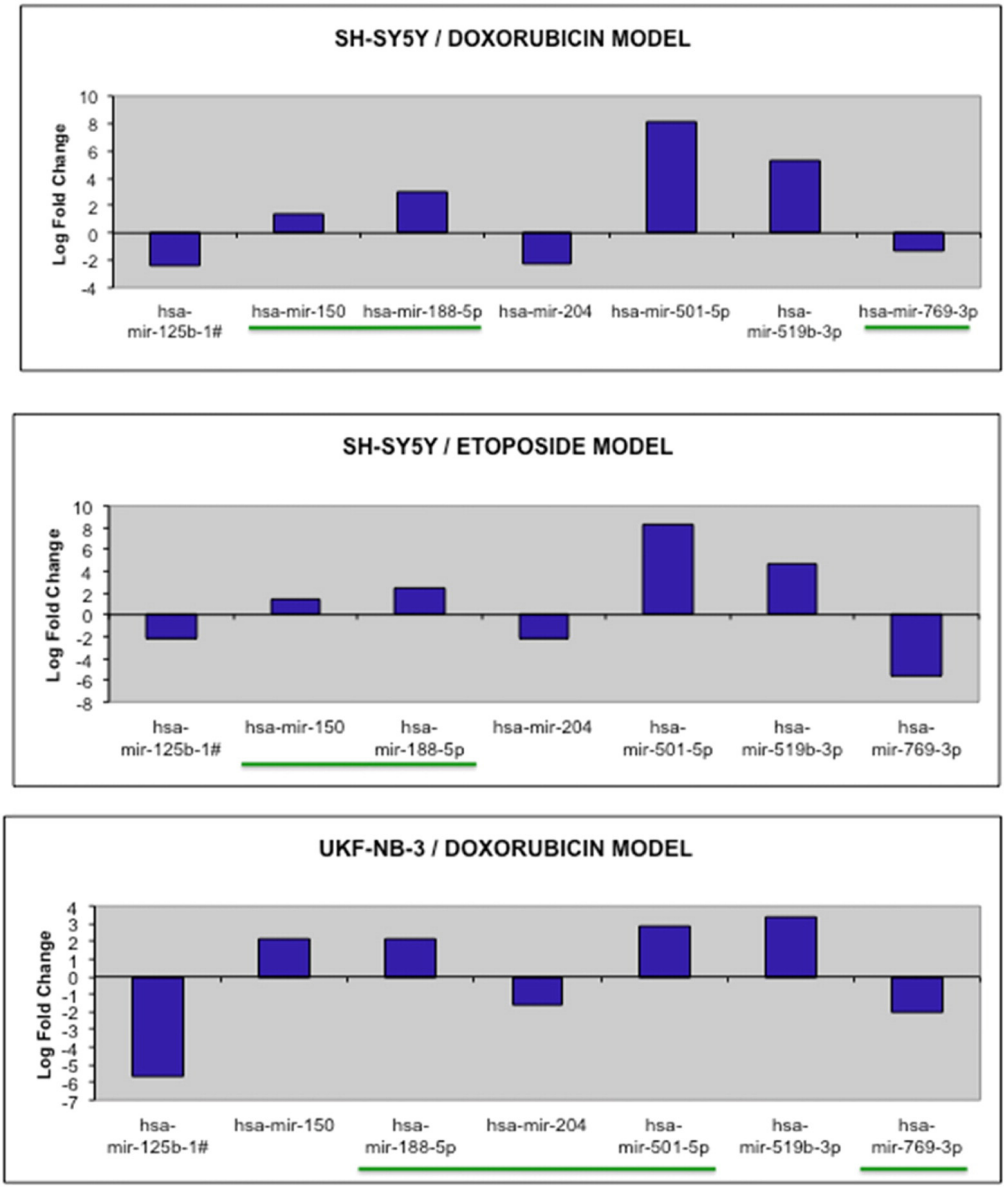
Graphs demonstrating log-fold change in miRNA expression for all three selected NB chemoresistance models. This miRNA signature of seven miRNAs was identified as dysregulated in the chemoresistant NB cell line components of all three selected NB chemoresistance models. miRNAs highlighted in green denote dysregulations in which both constituent cell lines had a raw Cq value below the 35 cutoff value. This specific Cq threshold value was utilised in order to eliminate false positive expression data inclusion due to quantification noise.

The results of the miRNA expression profiling revealed that a putative chemoresistance signature expression of seven miRNAs was present in all three NB chemoresistance cell line models (see Fig. 8). All seven miRNAs were consequently selected for validation of expression by multiplex RT-qPCR miRNA assay, prior to proceeding to chemoresistance function validation assays.

All identified miRNAs deemed to be involved in NB chemoresistance were consequently eligible for downstream validation studies.

### 3.3 Multiplex RT-qPCR for putative chemoresistance miRNAs

Once the short list of putative chemoresistance miRNAs was elucidated, it was deemed necessary to validate such findings by re-confirming the degree of dysregulated expression of each individual putative miRNA.

Since the miRNA profiling step did not utilise technical replicates for each individual miRNA RT-qPCR assay (one replicate/miRNA), it was essential to re-confirm the dysregulated expression status for each individual putative NB chemoresistance miRNA within each constitutive NB cell line of each chemoresistance model utilised in this study. This was implemented by performing a secondary RT-qPCR assay, with three technical replicates for each miRNA assay, in order to ascertain the specific miRNA expression levels.

**Fig. 9:**
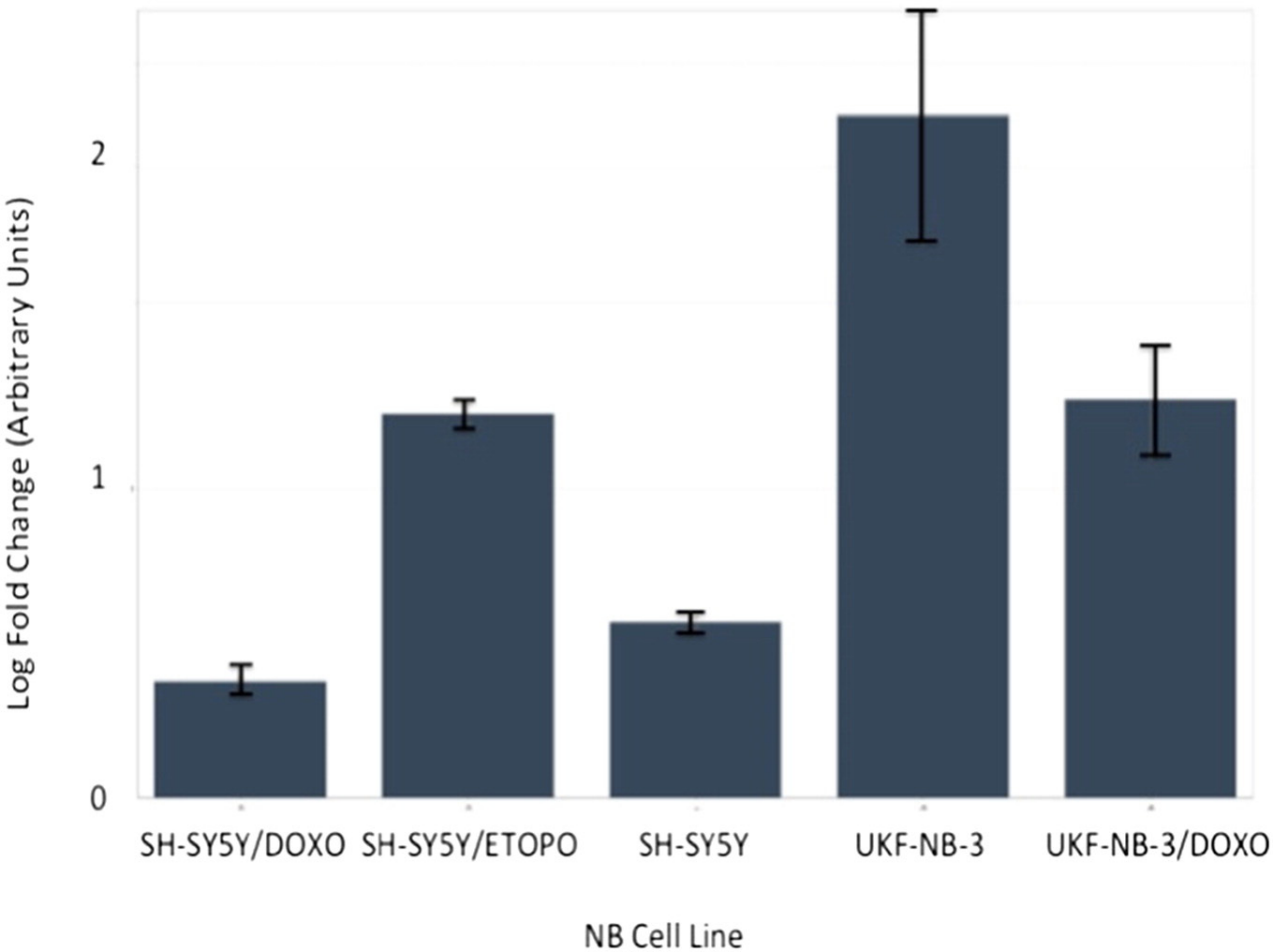
Multiplex RT-qPCR assay results (log-fold change) for hsa-miR-125b-1# following normalisation with reference miRNAs. Expected down-regulation of putative miRNA was confirmed in UKF-NB-3/DOXO and SH-SY5Y/DOXO models. Error bars represent standard deviation. Assays for hsa-mir-99b, hsa-mir-125a and hsa-mir-425 were also prepared as reference miRNAs for post-run data normalisation and analysis.

**Fig. 10:**
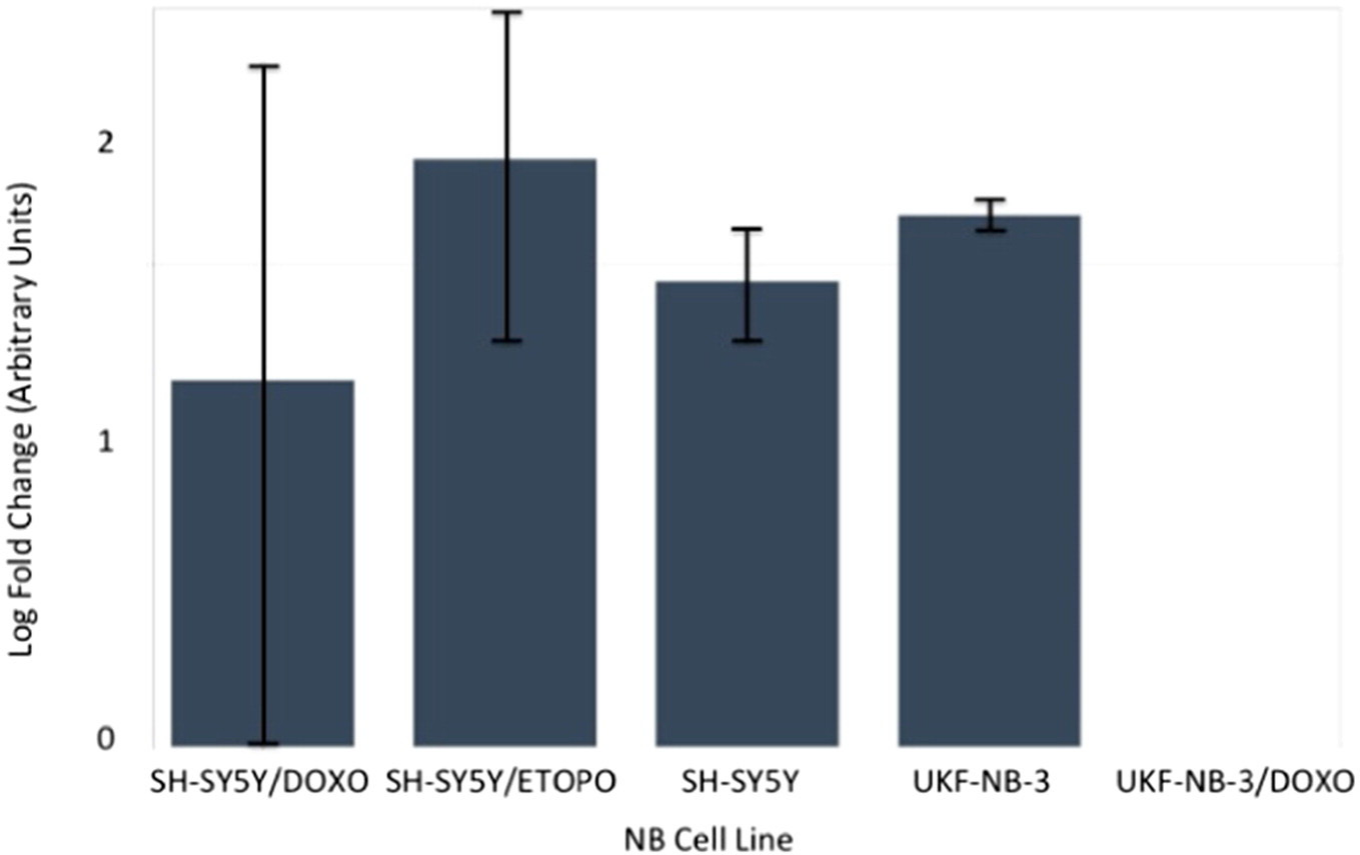
Multiplex RT-qPCR assay results (log-fold change) for hsa-miR-150 following normalisation with reference miRNAs. Expected up-regulation of putative miRNA was not confirmed in all three chemoresistance models. Error bars represent standard deviation. Assays for hsa-mir-99b, hsa-mir-125a and hsa-mir-425 were also prepared as reference miRNAs for post-run data normalisation and analysis.

**Fig. 11:**
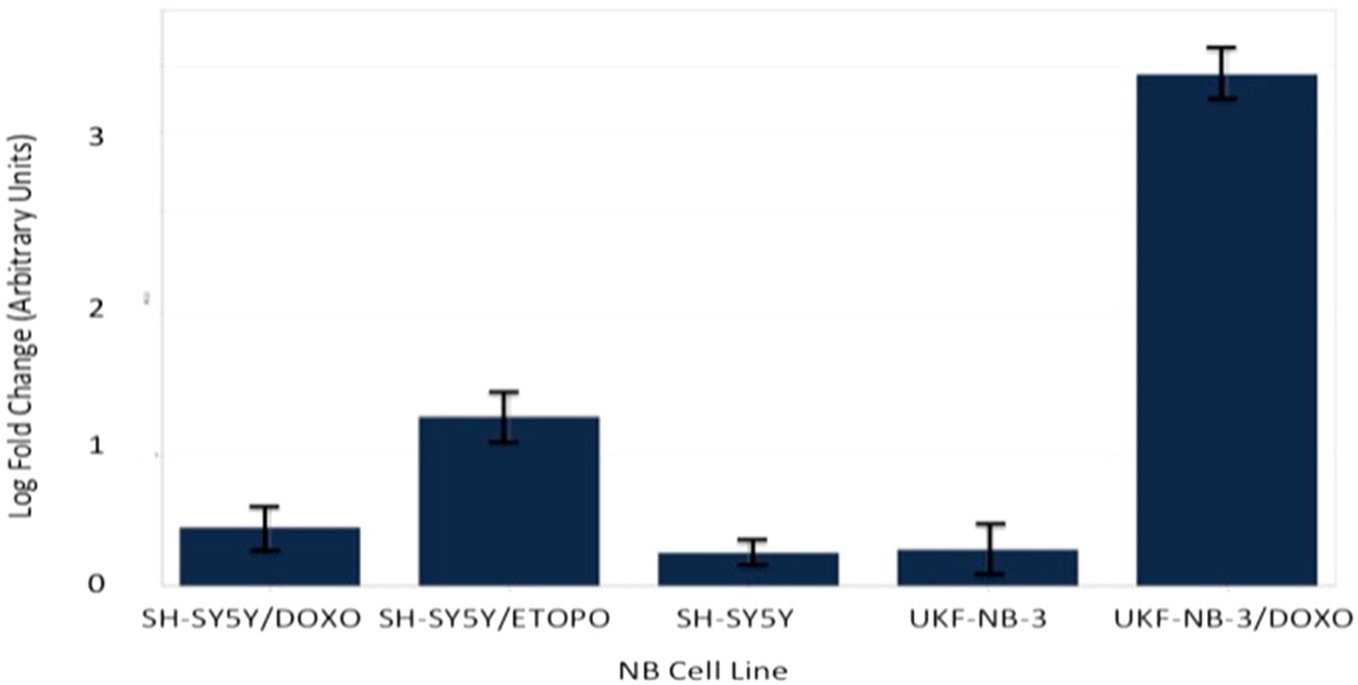
Multiplex RT-qPCR assay results (log-fold change) for hsa-miR-188-5p following normalisation with reference miRNAs. Expected up-regulation of putative miRNA was confirmed in all three NB chemoresistance models. Error bars represent standard deviation. Assays for hsa-mir-99b, hsa-mir-125a and hsa-mir-425 were also prepared as reference miRNAs for õpost-run data normalisation and analysis.

**Fig. 12:**
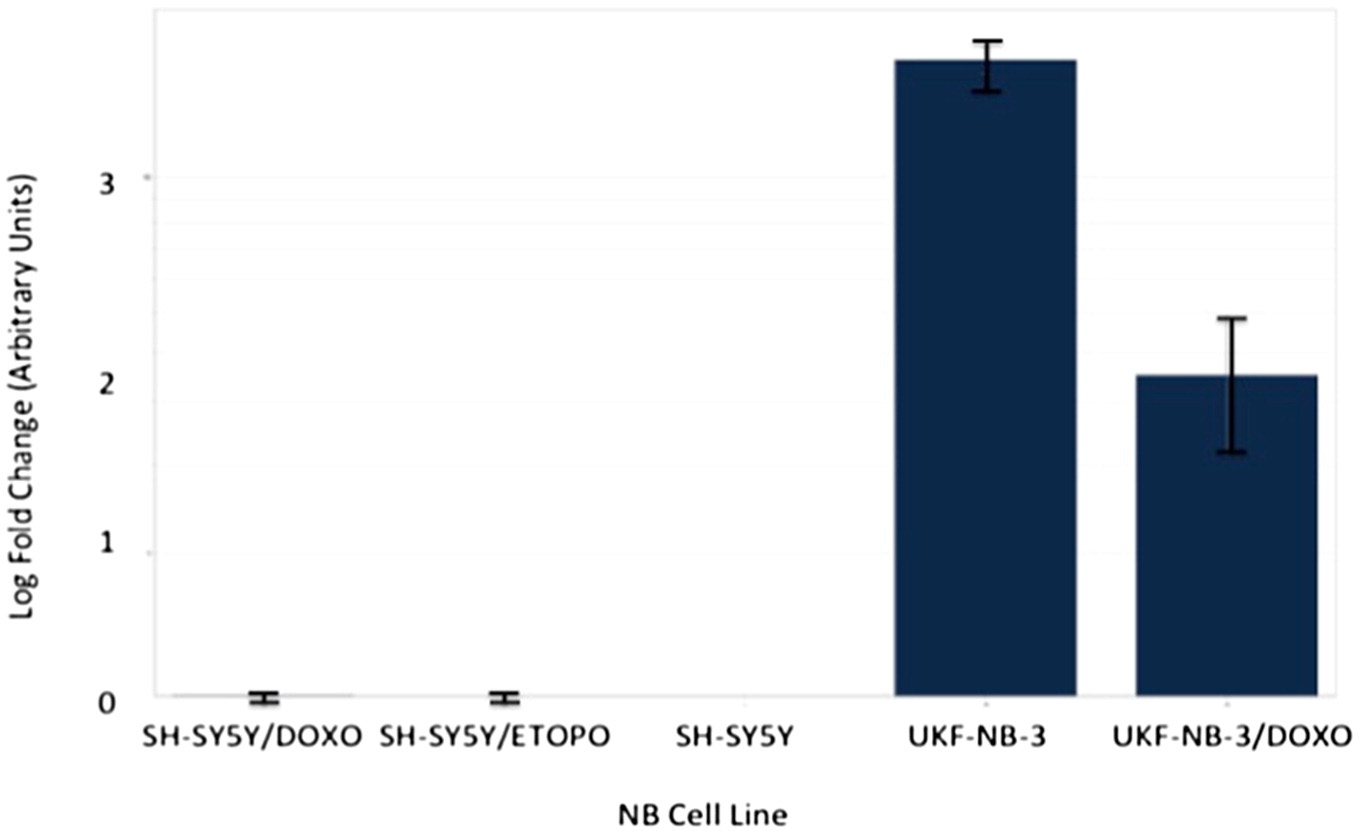
Multiplex RT-qPCR assay results (log-fold change) for hsa-miR-204 following normalisation with reference miRNAs. Expected down-regulation of putative miRNA was solely confirmed in UKF-NB-3/DOXO NB model. Error bars represent standard deviation. Assays for hsa-mir-99b, hsa-mir-125a and hsa-mir-425 were also prepared as reference miRNAs for post-run data normalisation and analysis.

**Fig. 13:**
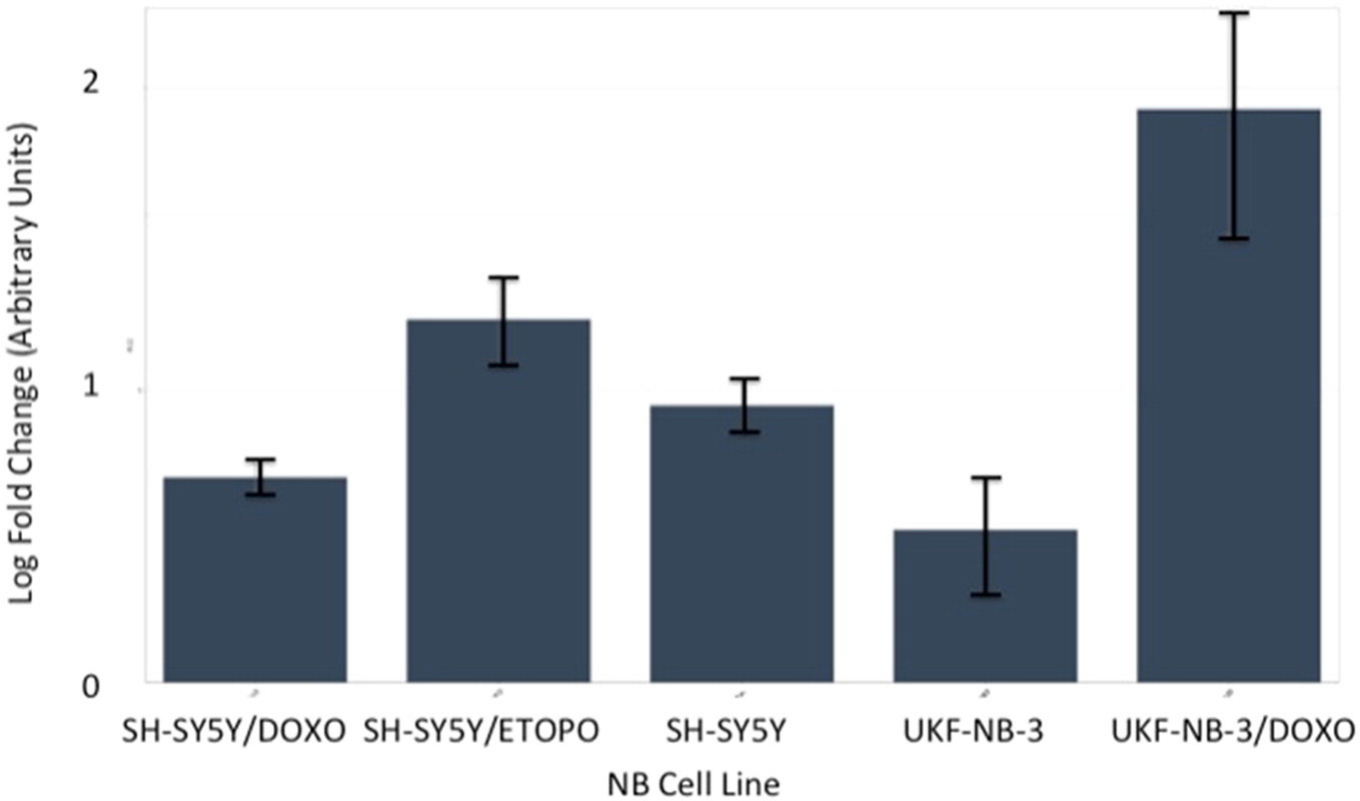
Multiplex RT-qPCR assay results (log-fold change) for hsa-miR-501-5p following normalisation with reference miRNAs. Expected up-regulation of putative miRNA was confirmed in UKF-NB-3/DOXO and SH-SY5Y/ETOPO models. Error bars represent standard deviation. Assays for hsa-mir-99b, hsa-mir-125a and hsa-mir-425 were also prepared as reference miRNAs for post-run data normalisation and analysis.

**Fig. 14:**
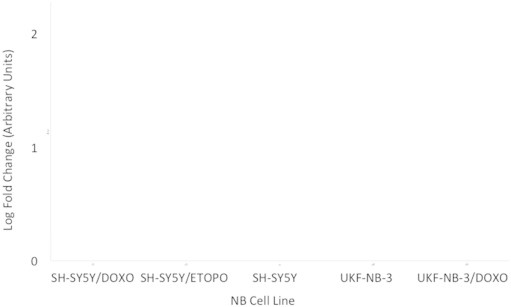
Multiplex RT-qPCR assay results (log-fold change) for hsa-miR-519b-3p following normalisation with reference miRNAs. Expected up-regulation of putative miRNA was not confirmed for all three chemoresistance models. Error bars represent standard deviation. Assays for hsa-mir-99b, hsa-mir-125a and hsa-mir-425 were also prepared as reference miRNAs for post-run data normalisation and analysis.

**Fig. 15:**
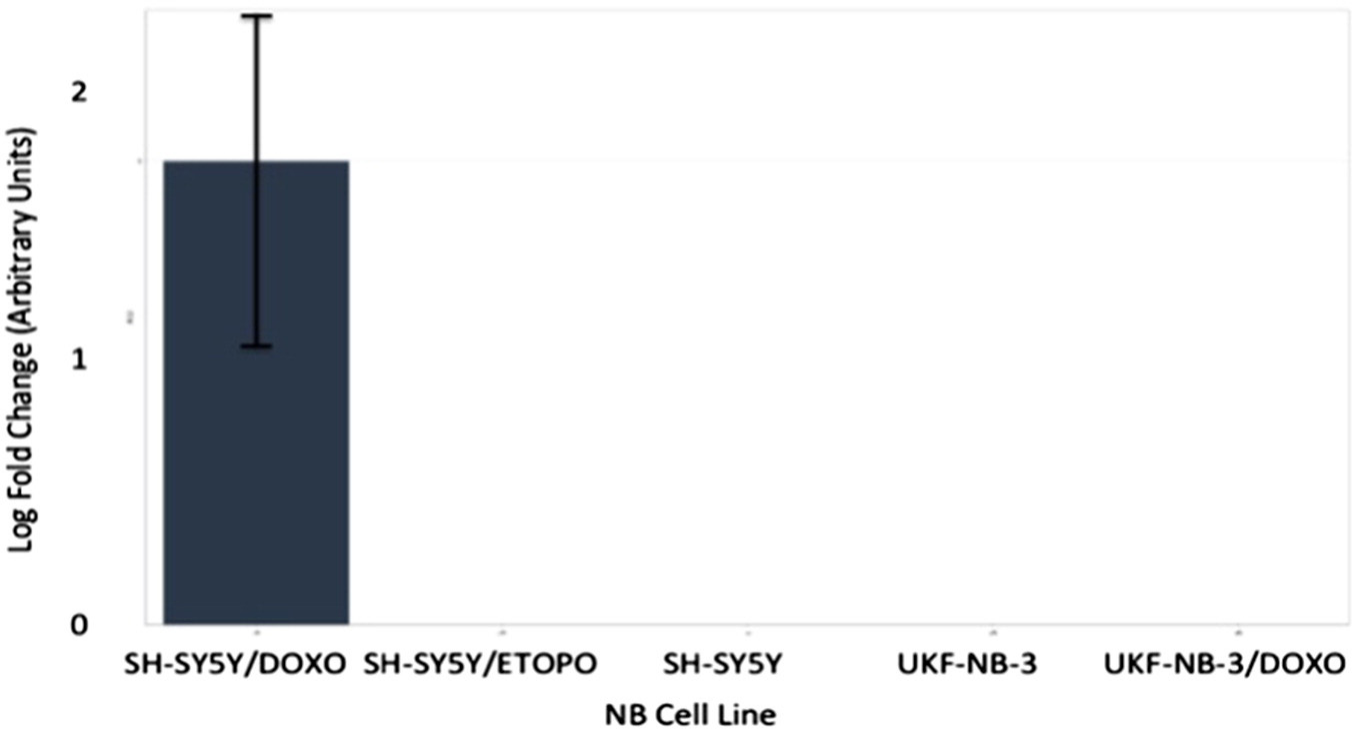
Multiplex RT-qPCR assay results (log-fold change) for hsa-miR-769-3p following normalisation with reference miRNAs. Expected down-regulation of putative miRNA was not confirmed in all three chemoresistance models. Error bars represent standard deviation. Assays for hsa-mir-99b, hsa-mir-125a and hsa-mir-425 were also prepared as reference miRNAs for post-run data normalisation and analysis.

Following re-analysis of RNA from each of the three NB chemoresistance models' cell line constituents by multiplex RT-qPCR miRNA assay, only four out of the original seven-member putative chemoresistance miRNA signature were pursued for functional validation (see Figs. 9–15).

## 4 Discussion

Initial performance results of the luminescence reader platform proved to be successful in achieving specificity of luminescence analysis, with a standard deviation of approximately 1%. Additionally, the dynamic range of the luminescence platform was also deemed optimal, with the lower end of the platform sensitivity spectrum denoted at a cell suspension density of 12,500 cells/mL.

From the eight NB chemoresistance cell line models at disposal in-house, only three models (SH-SY5Y/ETOPO, SH-SY5Y/DOXO and UKF-NB-3/DOXO) demonstrated distinct chemosensitivity profiles for the constitutive cell lines.

Analysis of the three Kelly NB chemoresistance models highlighted incongruencies in the chemosensitivity profiles of the component cell lines and was therefore excluded from the study.

For the UKF-NB-3 chemoresistance models, the UKF-NB-3/vincristine model was also excluded due to inability to confirm the projected chemosensitivity profiles for the two component cell lines, over two separate cell viability assays post-vincristine exposure. However, the UKF-NB-3/DOXO cell line reproducibly demonstrated to confirm the predicted chemosensitivity profiles, with the doxorubicin-resistant component cell line proving to be resistant to the drug exposure dose on comparison with the chemosensitive counterpart cell line by a factor of ten, on comparing the doxorubicin doses required to induce 50% decrease in cell viability. A peculiar observation was also denoted within the UKF-NB-3/DOXO model, in that at the elevated doxorubicin doses the cell viability in both component cell lines seems enhanced. This observation was found to be reproducible across two separate cell viability assays and was solely noticed within this specific NB chemoresistance model (UKF-NB-3 cell line exposed to doxorubicin). Possible explanations for such an anomaly would be technical interference with the luminescence assay, though the actual source thought to cause such interference is still unclear. Consequently, for the following transient transfection validation assays concerning the UKF-NB-3/DOXO model, the doxorubicin dose range utilised had to be reduced accordingly.

Analysis of the SH-SY5Y chemoresistance models revealed that the SH-SY5Y/cisplatin NB model had lost its chemoresistance phenotype, due to the observation of near-identical chemosensitivity profiles by both components of the NB model and therefore was excluded from the study. For the SH-SY5Y/DOXO and SH-SY5Y/ETOPO chemoresistance models, both met the predicted chemosensitivity profiles required for inclusion in the study, although it was also denoted that the SH-SY5Y/DOXO chemoresistance model demonstrated to be more robust. This was due to the enhanced chemoresistance properties highlighted by the doxorubicin-resistant cell line model component, on comparison with its etoposide-resistant counterpart cell line. Such phenotypes were confirmed for both NB chemoresistance models, following secondary cell viability assays.

The utilisation of RT-qPCR technology in a high throughput manner for the purpose of quantitatively analysing the miRnome in clinical and scientific studies is, of late, a rapidly developing tool for investigating miRNA expression. The essential reason for such popularity lies in the fact that such a method combines the high-throughput efficiency of the platform to simultaneously analyse almost 700 miRNAs from a single biological sample, though retaining the robustness and quantitative analytical advantage of RT-qPCR techniques. Such a technique was utilised in this study for identifying dysregulated expression of individual and/or networks of miRNAs within the selected NB chemoresistance cell line models.

Within the context of this study, pre-determined criteria were applied for the purpose of identifying putative chemoresistance miRNAs. All Cq values obtained for the investigated miRNAs, observed at a value above 35, would lead to the interpretation that such miRNAs were either not expressed at all, or expressed at very low levels in the corresponding biological sample. This specific Cq threshold value was thus utilised in order to eliminate false positive expression data inclusion due to quantification noise, which is normally introduced due to natural biological variances or also due to the technical setup of the RT-qPCR assay [24]. This is a fine balance that was applied to ensure that true amplification read-outs for miRNAs expressed at a low level (Cq value in the 30–35 range) would not be disregarded. Additionally, a defined threshold was applied for identifying which miRNAs in this study were deemed as putative chemoresistance miRNAs. Only the miRNAs that demonstrated linear fold change of above 2.0 (up-regulated) and below 0.5 (down-regulated) in the chemoresistant cell line component of each study model were included in the putative chemoresistance miRNA shortlist, on comparison of the normalised Cq values for each individual miRNA.

Such a fold change threshold range is deemed to be more restrictive as a selection strategy utilised for identifying relatively elevated levels of dysregulated expression by the individual miRNA. It is important to realise that such restrictive selection criteria might have excluded minor miRNA dysregulations (ie. less than two fold up-regulation or less than two-fold down-regulation) that may still prove to induce a tangible phenotypic influence (eg. fold-change × 1.8).

However, the fold change threshold was implemented in this study at such cut-off points for practical reasons, with the assumption that only those miRNAs falling within the dysregulation thresholds were deemed to have possible influence on NB cell line model chemoresistance properties.

Consequently, it must be emphasised that the threshold fold-change levels are entirely subjective and therefore a careful balance must be attained whereby reliable hits can be selected, though without elevating the stringency levels of the threshold fold-change values to the point at which identification of even a low quantity of hits would prove to be a challenge. Since the minimum miRNA fold change acceptable for scientific publications is a threshold of 1.5-fold, it would be recommended to set the least stringent miRNA fold-change selection criteria at this level (+/− 1.5 fold-change) for all studies of this nature [25–27].

The overview analysis of miRNA expression profiling of all three NB chemoresistance models demonstrated that approximately 50% of the miRNAs analysed in this study had a raw Cq value at/above a value of 35. Those miRNAs having a Cq value above the value of 35 were expressed at low/single molecule level and excluded from further analyses. The notion that half of all miRNAs are expressed at low levels (or not expressed) can be attributed to the fact that in most cancer conditions there exists an overall down-regulation of miRNA expression [28], with the overall percentage of miRNAs being expressed in normal, tumour-free tissues being higher than 50% (though such tissues were not analysed in this study).

In addition, the scatterplot analysis of normalised Cq values for each individual miRNA, obtained from each constitutive NB model component cell line, revealed overall low levels of data spreading. This implies that only a minute fraction of the entire miRnome may be deemed to be grossly dysregulated in such investigated cell line models.

For the three NB chemoresistance models investigated in this study and following implementation of the above selection criteria, a total of seven putative chemoresistance miRNAs were observed to be dysregulated across all three NB models (see Fig. 8). From such a shortlist of putative chemoresistance miRNAs, miR-188-5p demonstrated to have reliable RT-qPCR, due to all miR-188-5p dysregulations across all three NB chemoresistance models having Cq values below the 35.0 threshold. For the remaining six putative miRNAs, such an observation was not denoted from the RT-qPCR miRNA profiling analysis. This implies that for such miRNAs, in one or more NB cell line model, the dysregulated effect was deemed to be an ‘on/off’ effect, with the Cq value for the individual miRNA from one of the two NB cell line model components being above the pre-defined 35.0 value.

Following miRNA expression profiling, it was necessary to confirm dysregulation of the selected putative chemoresistance miRNAs by multiplex RT-qPCR techniques. This step was of importance for confirming the results obtained from miRNA profiling, since the latter RT-qPCR assays were performed using one sole technical replicate for each individual miRNA. Consequently, confirmation by multiplex RT-qPCR analysis utilising three technical replicates allowed for such validated putative chemoresistance miRNAs to be further validated by functional assay implementation.

Results from the multiplex RT-qPCR assays fully confirmed the up-regulated expression profile for miR-188-5p across all three NB chemoresistance models. Two additional putative miRNAs were also of major interest, namely miR-125b-1# and miR-501-5p, due to confirmation of dysregulated miRNA expression in at least two NB chemoresistance models. The fourth putative miRNA was miR-204. However, the down-regulated expression of miR-204 was only confirmed within the UKF-NB-3/DOXO chemoresistance model. This suggests that, due to the mutated p53 status present in UKF-NB-3, this dysregulation is cell line dependent and is totally absent in wild type p53 status NB cell lines such as SH-SY5Y [29].

The remaining miRNAs failed to confirm their dysregulated expression profile within the majority (or all) of the three NB chemoresistance models. The main reasons for such results stems from the RT-qPCR technique implemented for miRNA profiling. A typical step utilised for sample preparation prior to miRNA profiling is the pre-amplification of RNA collected from the biological sample [30].

Therefore, any miRNAs identified as expressed following miRNA profiling with a raw Cq value between 30 and 35 might eventually not be equally identified on multiplex/singleplex RT-qPCR analysis, since the latter is applied on non-pre-amplified RNA samples solely. Ultimately, such low expressed miRNA profiling expression values must be investigated with due attention and awareness of such technical influences [30].

Following the results of this study, exact mechanistic links between miRNA dys-regulation levels and chemoresistance phenotype cannot be concluded. However, it certainly is of interest to highlight the possible involvement of such miRNAs in affecting the most common means of chemoresistance-inducing cellular pathways within such cell lines. A typical example would be the effect of such miRNAs on the ABC transporter system and its member genes that regulate drug efflux properties of the cell [36].

Such mechanistic links have been identified within other cancer models. The study conducted by Ma and colleagues [37] demonstrated the key roles played by miR-133a and miR-326 in conferring adriamycin chemoresistance properties to the HepG2 hepatocellular carcinoma cell line model, through their regulatory effects on the ABCC1 gene. Furthermore, another study has highlighted the involvement of miR-27b in sensitising liver and kidney carcinoma cells to conventional cancer chemotherapies [38]. The study also demonstrated that miR-27b exerts its drug sensitivity influences through the activation of p53-dependent apoptotic pathways [38]. In colorectal cancer, miR-22 was recognised to affect drug sensitivity properties to 5-fluorouracil treatments through its involvement in the regulation of autophagy mechanisms, mainly due to miR-22 direct action on B-cell translocation gene 1 (BTG1) — a key molecular player in autophagy [39]. In gastric carcinoma, miR-129-5p CpG island methylations were identified to affect multi-drug resistance properties through targeted influences on ABC transporter genes [40].

Future research focusing on the influence of miRNA effects on NB chemoresistance properties may be highly varied and of equal importance. One specific research option would be to monitor the miRNA expression profile of individual NB cell lines during prolonged exposure to specific conventional chemotherapeutic agents, until such cell lines develop sustainable chemoresistance properties. Such a study would provide great insight onto which miRNA networks are most affected by prolonged cytotoxic drug exposure, through evaluation of miRNA profiles for dysregulated miRNA expression across the varying phases of the drug exposure period. Consequently, the miRNA profiles across the entire timeline for rendering a specific NB cell line chemoresistant to individual or combinations of chemotherapeutic agents may provide a wealth of information regarding which miRNAs could possibly have key roles in orchestrating such a phenotypic shift at the cellular level.

Furthermore, the prospect of performing miRNA profiling of NB cell lines having combinations of differing NB oncogene expression, as described above, could also shed more light on the effect of NB oncogene expression on miRNA expression profiles. Downstream miRNA dysregulations correlated to exacerbated oncogene expression may consequently be investigated for the identification and validation of key miRNAs deemed to act as intermediate effectors of chemoresistance.

The implementation of a systems biology approach might also be of valuable use for enhanced mapping and characterisation of miRNA networks with the capacity to interact and regulate complex gene networks directly affecting the various biological processes within the neuroblast that ultimately lead to the attainment of pronounced chemoresistance properties [31].

Ideally, should any miRNA have be successfuly validated within *in vitro* evaluations folowing our findings, further *in vivo* murine model research could be enacted, consisting of NB xenograft implants bearing constructs with individual putative chemoresistance miRNAs, which are actively expressed on oral administration of tetracycline by the individual mouse, thus minimising distress to the animal. Following miRNA induced expression, the animals would be exposed to cytotoxic agents and tumour progression is monitored accordingly. Induced expression of the putative chemoresistance modulating miRNA would be expected to lead to NB tumours having resilience to chemotherapy.

Clinical relevance of the identification of individual/networks of miRNAs directly linked with NB chemoresistance properties is two-fold.

Firstly, the identification and proper validation of hallmark miRNAs which are proven to be key players in conferring individual and/or multi — drug chemoresistance properties to NB tumours may be utilised for the development and implementation of novel diagnostic miRNA expression profile and screens [32]. These screens may prove essential for early recognition of NB tumours suspected to possess chemoresistance properties, thus giving the clinician an accurate picture of the clinical manifestation in the individual child suffering from NB. Following miRNA profiling and consequent functional assay validation techniques described above, the results are still inconclusive regarding adopting the seven-miRNA signature as hallmark biomarkers for NB chemoresistance. The results from the RT-qPCR study of oncogene over-expression on MDR gene dysregulation also proved to be inconclusive. Therefore, future studies such as the experimental designs suggested for future research described above might help to identify other miRNAs with more reliable and robust validation assay results to confirm their involvement in the induction of NB chemoresistance properties. Such identified miRNAs would ultimately be utilised in the clinical setting for diagnostic purposes to recognise NB tumours having chemoresistance properties to conventional chemotherapeutic agents.

Similar diagnostic screens have already been designed for accurate diagnosis of other severe conditions such as human papilloma viral infectious strains, though such bespoke microarray technology could be implemented for the recognition of specific miRNA sequences present in any clinical sample [33]. For NB diagnostic purposes, identification of novel biomarkers may also be attained through the profiling of other classes such as long non-coding RNA molecules (utilising RT-qPCR), whole genome microarray profiling and also exome next-generation sequencing.

However, the validation methods for confirming individual candidate biomarkers may be time consuming. This is a particular problem for exome sequencing approaches, due to the vast array of data that is commonly generated and thus requires thorough bioinformatic analysis prior to even identifying candidate exome sequence biomarkers for consequent validation assays. In distinct contrast, the possibility of identifying novel body fluid biomarkers, such as free-circulating miRNAs [34], ultimately provides an extremely rapid diagnostic/prediction test within the clinical setting due to the sole requirement of a blood sample for RT-qPCR analysis. However, biomarker validation techniques are very much dependant on the specific phenotype with which the individual candidate biomarker needs to be reliably linked to (*e.g.* chemoresistance, cell proliferation, cell differentiation).

For the validation of miRNAs associated with chemoresistance, transient transfection of miRNA antagonists remains the mainstay method due to the rapidity of the assay (72–96 h). Unfortunately, this method also has the drawback that direct quantification of candidate biomarker miRNA by RT-qPCR cannot be performed, unless the miRNA in question is known to down-regulate specific target genes (which have been previously confirmed in other studies).

Following the acquisition of additional information from these novel biomarkers, the therapeutic avenues chosen by the clinician will be decided with the inclusion of pre-emptive strategies against chemoresistant tumours, based on the additional awareness of the chemosensitivity status of the individual NB tumour. The therapeutic options applied will thus maximise the chances of survival of the patient, with minimal suffering due to chemotherapy-induced adverse effects.

Secondly, such miRNAs are good candidates to be novel drug targets for future RNAi based therapies against aggressive tumours that are not responding to conventional chemotherapy [35]. These miRNA-directed therapies are still in their infancy, though may in future prove to be a novel molecular approach aimed at enhancing the efficacy of conventional chemotherapeutic treatments, with possible reduction in the dose and frequency of chemotherapy cycles through which the child has to undergo, ultimately reducing the level of distress and suffering from chemotherapy-induced adverse effects.

## Figures and Tables

**Table 1:** List of NB chemoresistance cell line models. Subcultures of each parental cell line were induced to acquire chemoresistance properties towards a single chemotherapeutic agent. The SH-SY5Y and Kelly cell lines were obtained from the Eggert group, Essen, Germany. The UKF-NB-3 cell lines were obtained from the Cinatl Jr group, Frankfurt, Germany.

NB cell line	Chemotherapeutic agent
SH-SY5Y	Cisplatin
SH-SY5Y	Doxorubicin
SH-SY5Y	Etoposide
KELLY	Cisplatin
KELLY	Doxorubicin
KELLY	Etoposide
UKF-NB-3	Doxorubicin
UKF-NB-3	Vincristine

**Table 2:** Chemotherapeutic drug ranges utilised for investigating chemosensitivity status in each cell line. All treatments were performed in triplicate for each drug dose.

Chemotherapeutic Drug dose range (ng/mL)				
Plate row location	Cisplatin	Doxorubicin	Etoposide	Vincristine
B	50,000	28,999	1,000,000	4615
C	5000	2899.9	100,000	461.5
D	500	289.99	10,000	46.15
E	50	28.999	1000	4.615
F	5	2.9	100	0.465
G	0.5	0.29	10	0.046
